# Effect of cooking locally available common bean (*Obwelu*) on iron and zinc retention, and pumpkin (*Sweet cream*) on provitamin A carotenoid retention in rural Uganda

**DOI:** 10.1002/fsn3.1873

**Published:** 2020-09-20

**Authors:** Edward Buzigi, Kirthee Pillay, Muthulisi Siwela

**Affiliations:** ^1^ Department of Dietetics and Human Nutrition School of Agricultural, Earth and Environmental Sciences University of KwaZulu‐Natal Pietermaritzburg South Africa; ^2^ Health Economics and HIV/AIDS Research Division (HEARD) University of KwaZulu‐Natal Durban South Africa; ^3^ Department of Human Nutrition & Home Economics Kyambogo University Kampala Uganda

**Keywords:** common bean, cooking, iron, provitamin A carotenoids, pumpkin, retention, zinc

## Abstract

Pumpkin is a potential rich source of vitamin A precursors called provitamin A carotenoids (PVACs), while common bean is a potential rich source of iron and zinc. This study evaluated the effect of cooking locally available pumpkin, *Sweet cream* (*Cucurbita moschata*) on PVACs retention in Uganda. Furthermore, the effect of cooking locally available common bean, *Obwelu* (*Phaseolus vulgaris*) on iron and zinc retention was evaluated. Expert caregivers from the local community cooked pumpkin by either boiling or steaming, while common bean was cooked by either boiling with prior soaking or boiling without prior soaking. PVACs in raw and cooked pumpkin were analyzed by high‐performance liquid chromatography (HPLC), while iron and zinc in raw and cooked common bean were analyzed by flame atomic absorption spectroscopy (FAAS). Conversion of PVACs into vitamin A retinol activity equivalents (RAE) was calculated using the Institute of Medicine (2001) recommendations for the bioconversion of PVACs into vitamin A. Micronutrient retention was measured using true retention. β‐carotene, α‐carotene, and vitamin A content in raw pumpkin was 1,704 µg/100 g, 46 µg/100 g and 1,437 µgRAE/100 g, respectively. Either boiling or steaming pumpkin resulted in over 100% retention of PVACs and vitamin A. Iron and zinc retention for boiled common bean with prior soaking was 92.2% and 91.3%, respectively. Boiling common bean without prior soaking resulted in 88.4% and 75.6% retention of iron and zinc, respectively. In conclusion, to retain a high proportion of PVACs caregivers should be advised to cook *Sweet cream* by either boiling or steaming, while to retain a high proportion of iron and zinc, *Obwelu* should be prepared by boiling with prior soaking.

## Graphical abstract

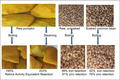

## 1 INTRODUCTION

The consequences of vitamin A deficiency (VAD), iron deficiency (ID), and zinc deficiency (ZnD) among children are debilitating because they are associated with childhood mortality and morbidities such as night blindness, diarrhea, iron deficiency anemia, and stunting, respectively (Millward, 2017). In rural Uganda, children aged 6 to 24 months are at a higher risk of developing VAD, ID, and ZnD because they are fed complementary foods (CFs) predominantly formulated from staple cereals such as white maize and tubers such as white cassava, white sweet potato and yams (Amaral, Herrin, & Gulere, 2018; Ekesa, Nabuuma, & Kennedy, 2019). These staples are deficient in vitamin A, iron, and zinc or contain antinutrients that reduce the bioavailability of zinc and iron (Gibson, Bailey, Gibbs, & Ferguson, 2010). Hence, their predominance in the diets of children increases the risk for VAD, ID, and ZnD (Gegios et al., 2010).

To combat child VAD, ID, and ZnD, the World Health Organization (2009) recommends strategies such as micronutrient supplements, commercially fortified foods, and animal source foods (ASFs) to prepare CFs for children in the age range of complementary feeding. This is plausible because micronutrient fortified foods, supplements, and ASFs are rich sources of vitamin A, iron, and zinc. However, rural caregivers in Uganda cannot afford food supplements, commercially fortified foods and ASFs (Wamani, 2005). Therefore, rural caregivers resort to using locally available vitamin A, iron and zinc‐deficient CFs prepared from vitamin A, iron and zinc‐deficient staples cereals and tubers (Ekesa et al., 2019), which increases child vulnerability to VAD, ID, and ZnD. Locally available pumpkin species in Uganda such as *Cucurbita moschata* and *Cucurbita maxima* (Ondigi et al., 2008; Nakazibwe, 2019), are potential sources of provitamin A carotenoids (PVACs) such as β‐carotene, α‐carotene, and β‐cryptoxanthin (Kim, Bukenya‐Ziraba, Namaganda, & Mulumba, 2012; Koh & Loh, 2018; Saini, Nile, & Park, 2015). Moreover, when PVACs‐rich foods are consumed, the human body bioconverts these PVACs into retinol, the active form of vitamin A used by the body (Van Loo‐Bouwman, Naber, & Schaafsma, 2014). Furthermore, common bean (*Phaseolus vulgaris*) is locally available in Uganda, and it is a potential rich source of iron and zinc (Beebe, Gonzalez, & Rengifo, 2000; Carvalho et al., 2012; Kiwuka, Bukenya‐Ziraba, Namaganda, & Mulumba, 2012).

To contribute toward improving the vitamin A, iron, and zinc nutrient density of complementary foods in Uganda (Ekesa et al., 2019), locally available PVACs‐rich pumpkin (*Sweet cream*), and iron and zinc‐rich common bean (*Obwelu*) were identified from the local community to prepare a PVACs, iron and zinc‐rich CF in rural Uganda (Buzigi, Pillay, & Siwela, 2020). It is worth noting that raw foods should be prepared by cooking to make them palatable and soft for children to consume as CFs (FAO, 2017). However, nutrient retention, defined as the ratio of nutrient content in the cooked food to the nutrient content in the raw food is affected by different cooking methods (Li, Tayie, Young, Rocheford, & White, 2007). Several cooking methods impact differently on PVACs retention in PVAC‐rich foods including different pumpkin varieties (Azizah, Wee, Azizah, & Azizah, 2009; Bechoff, 2017; Bengtsson, Namutebi, Alminger, & Svanberg, 2008; Carvalho et al., 2015; Carvalho, Smiderle, Carvalho, Cardoso, & Koblitz, 2014; De Moura, Miloff, & Boy, 2013; Mugode et al., 2014). Similarly, cooking impacts differently on iron and zinc retention in common bean varieties (Carvalho et al., 2012; Ferreira, Naozuka, Kelmer, & Oliveira, 2014). However, the effect of cooking pumpkin, *Sweet cream* on PVACs retention and the effect of cooking common bean, *Obwelu* on iron and zinc retention is unknown. To this end, this study evaluated the effect of household cooking methods used in rural Uganda on the retention of PVACs in pumpkin (*Sweet cream*) and iron and zinc retention in common bean (*Obwelu*). The cooking methods found to retain a high proportion of micronutrients will be recommended for use while preparing pumpkin and common bean in Uganda.

## 2 MATERIALS AND METHODS

### 2.1 Pumpkin and common bean materials

Pumpkin (*Sweet cream*) and common bean (*Obwelu*) were purchased from the local market in rural Kyankwanzi district, central Uganda.

### 2.2 Selection of home cooking methods for common bean and pumpkin

Pumpkin and common bean were prepared using the local household‐level cooking methods as recommended by the 2017 Food and Agriculture Organization of the United Nations (FAO) guide to conducting participatory cooking demonstrations to improve complementary feeding practices (FAO, 2017). To this end, this study worked with Village Health Team members (VHTs) and peer mothers from the study community. The Ugandan Ministry of Health (MOH) recognizes VHTs as community health workers at the village level with the roles of promoting, supporting, and protecting community health programs such as nutrition and health in their respective villages (MOH, 2016). By using simple random sampling, the head of VHTs identified one expert peer mother from each of the 10 villages located in Ntwetwe sub county, Kyankwanzi district, Uganda to participate in the cooking of pumpkin and common bean, using household‐level cooking methods of common bean and pumpkin as used in the study community.

**FIGURE 1:**
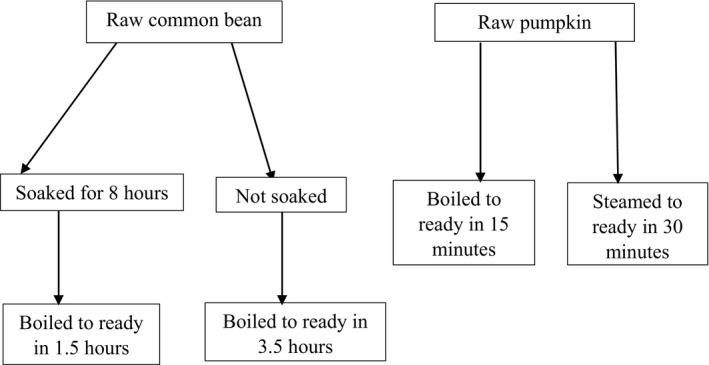
Flow chart of methods used to cook common bean and pumpkin

Expert peer mothers were asked one by one to mention the cooking methods commonly used at household level to cook common bean and pumpkin in their community. Of the 10 expert peer mothers, 9 and 10 mentioned overnight soaking followed by boiling and boiling without soaking, respectively as the commonly used household cooking methods for preparing common bean in their community. Furthermore, of the 10 expert peer mothers, 9 and 8 mentioned that pumpkin was commonly cooked by boiling and traditional steaming, respectively. To this end, PVACs retention in pumpkin was evaluated either by boiling and steaming pumpkin, while iron and zinc retention in common bean was evaluated by boiling with or without overnight soaking. Figure 1 shows the flow chart of methods used to cook pumpkin and common bean.

### 2.3 Preparation and cooking of pumpkin

Expert peer mothers participated in the preparation and cooking of the study pumpkin and common bean. Pumpkin pulp was washed, cut into three relatively equal sections, and seeds discarded. One raw section was peeled and blended for about 1 min using a blender to obtain a homogenous sample for PVACs analysis. The other sections were cooked according to one of the two following procedures commonly used by households in the community, as indicated by expert peer mothers. The first section was peeled and further divided into four relatively equal parts. The four parts were then put into a cooking pot, and tap water was added to the pumpkin level. The pot with a lid was transferred to a hot charcoal stove and boiled for about 15 min. The second section was also cut into four relatively equal parts, peeled, and made ready for a local method of steaming.

**FIGURE 2:**
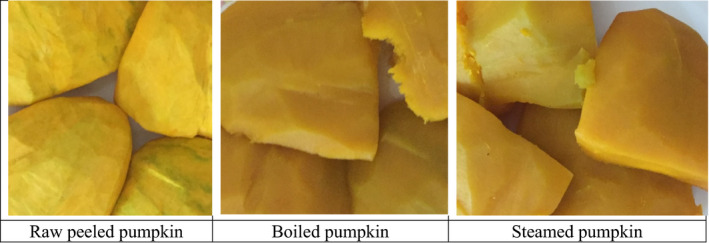
Raw and cooked pumpkin,*Sweet cream*

The local method of steaming involved putting 1 liter of tap water into the cooking pot, followed by banana stalks, then banana leaves, in which pumpkin was wrapped before being put onto the hot charcoal stove for steaming. The role of the banana stalk was to create an elevation and separation between the water in the cooking pot from the banana leaves, where the pumpkin was wrapped. To this end, when water in the pot boils, it releases steam that vaporizes through the banana stalk spaces to heat the banana leaves in which the pumpkin is wrapped. Pumpkin was steamed for about 30 min. Figure 2 shows raw and cooked pumpkin.

The durations of boiling and steaming were sufficient to soften the plant tissue as assessed by the penetration of the tip of a knife without resistance (Carvalho et al., 2014). Boiled and steamed pumpkin were blended separately in a blender for approximately 1 min to form separate homogenous samples of boiled and steamed pumpkin, ready for PVACs analysis.

### 2.4 Preparation and cooking of common bean

**FIGURE 3:**
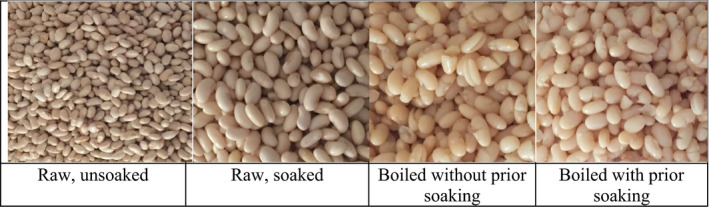
Raw and cooked common bean, *Obwelu* used in the study

1,000 g of common bean was divided into two portions, that is, 200 g (raw) and 800 g (for cooking). Raw common bean was dried in an oven at 80°C for 8 hr. Thereafter, it was milled to form a fine powder and kept in an airtight polythene bag ready for analysis. The remaining 800 g of common bean was further divided equally into two portions of 400 g each. The first portion was put into a cooking pot and soaked in tap cold water overnight (8 hr). The water was discarded in the morning, and the soaked common bean was transferred to the regular cooking pot. The second portion was washed in cold tap water and transferred to the regular cooking pot. The cooking pots were filled with tap water to cover just above the level of the common bean in the pot. A lid was placed on each pot prior to putting it on the hot charcoal stove ready for boiling. The expert peer mothers regularly checked on the common bean to confirm whether it was soft and ready for consumption. When it was established that it was ready, common bean was taken off the charcoal stove. Figure 3 shows raw and cooked common bean.

The cooking time for boiled common bean with and without soaking was 1.5 and 3.5 hr, respectively. Thereafter, the water used to boil the common bean was drained, and the common beans were put in a blender and blended for around 1 min ready for analysis.

### 2.5 Analysis of provitamin A carotenoids and retinol activity equivalent in pumpkin

The PVACs content was analyzed by high‐performance liquid chromatography (HPLC) as described in the HarvestPlus handbook for carotenoid analysis (Rodriguez‐Amaya & Kimura, 2004). Prior to PVACs identification and quantification by HPLC, carotenoids were extracted from the pumpkin. For the carotenoid extraction, 25 ml of acetone was successively added to 1.0 g of blended freeze‐dried pumpkin (separate for raw, boiled, and steamed), until a paste was obtained. The paste was transferred to a sintered funnel (5 µm) coupled to a 250 ml Buchner flask and filtered. This procedure was repeated at least three times until the sample was colorless. The obtained extract was transferred to a 500 ml separatory funnel containing 40 ml of petroleum ether. The acetone was removed through the slow addition of ultrapure water (Milli‐Q‐Millipore) to prevent the formation of an emulsion. The aqueous phase was discarded, and this procedure was repeated four times until no residual solvent remained.

For the identification and quantification of PVACs, 2 ml were removed from the carotenoid extract for raw, boiled, and steamed pumpkin and dried in an amber flask under flowing nitrogen. The sample was diluted in 100 µl of acetone, while mixing in a vortex mixer and then transferred to a 2 ml amber flask for HPLC analysis. All the solvents and chemicals including α‐carotene and β‐carotene standards were purchased from valid sources. Calibration curves were used to calculate the concentration of the respective carotenoids. The content of α‐carotene and β‐carotene was calculated according to the formula:

$$C\left(\mu g/g\right)=\frac{A_{X}\times C_{S}\left(\mu /g\right)\times V\left(\text{ml}\right)}{A_{S}\times P\left(g\right)}$$

Where: *A_X_* = Carotenoid peak area; *C_S_* = Standard concentration; *A_S_* = Standard area; *V* = Total extract volume and *P* = Sample weight.

### 2.6 Conversion of provitamin A carotenoids in pumpkin to vitamin A (retinol activity equivalent)

The Institute of Medicine (2001) bioconversion rates of PVACs to retinol (retinol activity equivalents) were used; that is, 12 µg of β‐carotene is equivalent to 1 µg of retinol, while 24 µg of α‐carotene is equivalent to 1 µg retinol (Institute of Medicine, 2001).

### 2.7 Analysis of iron and zinc in common bean

Iron and zinc concentrations for both raw and boiled common bean were determined by flame atomic absorption spectroscopy (FAAS) as described previously (Ekesa et al., 2019; Santelli, de Almeida, de SantAna, Cassella, & Ferreira, 2006). Microwave digestion was used to dissolve iron and zinc to oxidize the organic molecules in common bean prior to analysis by FAAS. Microwave digestion was done by closed vessel microwave oven following the procedure by Santelli and colleagues (Santelli et al., 2006). 0.5 g of each sample was carefully weighed into perfluoroalkoxy (PFA) liners of the oven and 8 ml of concentrated nitric acid was added. After standing overnight in contact with nitric acid, 2 ml of 30% m/m hydrogen peroxide was added and the liners were closed, placed into the microwave oven cavity and two irradiation cycles of 25 min (1,000 W maximum power) were run. The program of the equipment was adjusted to avoid internal pressures higher than 180 psig and temperatures higher than 190°C and between the first and second cycle, the liners were opened to release gases formed during digestion. Applying this procedure, perfectly clear and colorless solutions were obtained. This solution was transferred to the volumetric flask, and the volume was made up to 25 ml. This final solution was used in the iron and zinc determination by FAAS. The sample solution was then transformed into aerosols and transported to the flame to vaporize and atomize the sample. This step led to a reduced intensity of the light (by absorption of a defined quantity of energy) coming from a hollow‐cathode lamp. The attached detector quantified the absorbed incoming light. The difference between the radiation without sample and including sample (absorbance) was used to calculate the iron and zinc concentration.

### 2.8 Retention of provitamin A carotenoids, iron, and zinc

Micronutrient retention in processed foods may be calculated as either apparent retention (AR) or true retention (TR) (Li et al., 2007; Murphy, Criner, & Gray, 1975). Apparent retention assumes that the amount of solids lost during processing are negligible and is expressed on a dry weight basis (Li et al., 2007; Murphy et al., 1975). In contrast, true retention is expressed on a fresh weight basis (Li et al., 2007). It is worth noting that TR considers the loss of physical mass (i.e., soluble solid losses) more especially in food processing methods such as cooking, which cause significant soluble solid losses (Bechoff et al., 2017). Therefore, TR is more accurate than AR (Bechoff et al., 2017). To this end, micronutrient retention of PVACs, iron and zinc was calculated by TR using the equation below (Bechoff et al., 2017).

$$\text{TR}\left(\%\right)=\frac{M_{2}C_{2\;}\times 100}{M_{1}C_{1}}$$

Where TR is true retention, *M*_2_ is mass of cooked food in grams, *C*_2_ is micronutrient content per gram of cooked food, *M*_1_ is mass of raw food in grams and *C*_1_ is micronutrient content per gram of raw food.

### 2.9 Data analysis

Data for iron and zinc content were reported as mean (mg/100 g) of dry fresh weight ± standard deviation (*SD*) of three determinations for raw and boiled common bean with or without prior soaking. The PVACs were reported as (µg/100 g) fresh weight ± *SD* of three determinations for raw, boiled, and steamed pumpkin. Vitamin A, retinol was reported as retinol activity requirements (RAE) measured in µg/100 g for raw, boiled, and steamed pumpkin. Data analysis was done using statistics and data (STATA), version 13.1.

## 3 RESULTS

The aim of the study was to determine the effect of household cooking methods on PVACs retention in locally available pumpkin (*Sweet cream*) in Uganda. Furthermore, it evaluated the effect of household cooking methods on zinc and iron retention in common bean (*Obwelu*) in Uganda.

### 3.1 Effect of cooking pumpkin on provitamin A carotenoid

Pumpkin was cooked by either boiling or steaming. Boiling and steaming pumpkin took 15 and 30 min, respectively. Table 1 shows the PVACs content and retention after boiling and steaming pumpkin.

**TABLE 1:** Provitamin A carotenoid content and retention in boiled and steamed pumpkin, *Sweet cream* in Uganda

PVACs	Raw	Boiled	Steamed	True retention (%)	
				Boiled	Steamed
β‐carotene µg/100 g (±*SD*)	1,704 (±5.0)	3,326.5 (±0.7)	3,466.2 (±3.9)	195	203
α‐carotene µg/100 g (±*SD*)	46 (±3.0)	75.1 (±0.3)	88.0 (±0.6)	161	194
Vitamin A, (RAE) µg/100 g (±*SD*)	143.9 (±4)	280.3 (± 0.8)	292.5 (± 2)	194.8	203.3

Note

Observations of PVACs and retention are given as a mean of three determinations. RAE = β‐carotene (µg/100 g)/12 + α‐carotene (µg/100 g)/24 (Institute of Medicine, 2001).

PVACs, Provitamin A carotenoids; RAE, Retinol Activity Equivalents, Vitamin A (retinol); *SD*, Standard deviation.

Mean β‐carotene content was higher in boiled (3,326.5 µg/100 g) and steamed (3,466.2 µg/100 g) pumpkin than in raw pumpkin (1,704 µg/100 g). Furthermore, mean β‐carotene content was higher in steamed compared to boiled pumpkin. The TR for β‐carotene in boiled and steamed pumpkin was 195% and 203%, respectively. Mean α‐carotene was higher in boiled (75.1 µg/100 g) and steamed pumpkin (88 µg/100 g) compared to raw pumpkin (46 µg/100 g). It is worth noting that the mean α‐carotene content was higher in steamed pumpkin compared to boiled pumpkin. The TR for α‐carotene in boiled and steamed pumpkin was 163% and 191%, respectively. The RAE was calculated by using the 2001 Institute of Medicine bioconversion rates for PVACs to retinol (Institute of Medicine, 2001). The calculated mean RAE was higher in boiled pumpkin (280.3 µg/100 g) and steamed pumpkin (292.5 µg/100 g) than in raw pumpkin (143.9 µg/100 g). The TR for RAE in boiled and steamed pumpkin was 194.5% and 203.3%, respectively.

### 3.2 Effect of cooking common bean on iron and zinc retention

Common bean was cooked either by soaking followed by boiling or boiling without prior soaking. Common bean was cooked until they were soft and ready for human consumption. The cooking time for common bean which was soaked first was 1.5 hr, while common bean which was not soaked took 3.5 hr to cook. Table 2 shows iron and zinc retention of boiled common bean in rural Kyankwanzi district, Uganda.

**TABLE 2:** Iron and zinc retention of cooked common bean, *Obwelu* in Uganda

Cooking method	True retention (%)	
	Iron	Zinc
Boiling with prior soaking	92.2	91.3
Boiling without soaking	88.4	75.6

The mean iron content reduced from 7.75 mg/100 g in raw common bean to 7.13 mg/100 g in boiled common bean with prior soaking, giving an iron retention of 92.2%. Likewise, mean zinc content reduced from 3 mg/100 g in raw common bean to 2.73 mg/100 g in boiled common bean with prior soaking, giving a zinc retention of 91.3%. Furthermore, mean iron content reduced from 7.75 mg/100 g in raw common bean to 6.84 mg/100 g in boiled common bean without prior soaking, giving an iron retention of 88.4%. Furthermore, mean zinc content reduced from 3 mg/100 g in raw common bean to 2.26 mg/100 g in boiled common bean without prior soaking, giving a zinc retention of 75.6%.

## 4 DISCUSSION

The two methods that can be used to calculate nutrient retention are AR and TR (Li et al., 2007; Murphy et al., 1975). However, the current study used TR because it is more accurate than AR since AR tends to overestimate nutrient retention (Bechoff et al., 2017; Hummel et al., 2020; Murphy et al., 1975).

### 4.1 Provitamin A carotenoids retention in pumpkin, *Sweet cream*

The higher concentrations of PVACs observed in boiled and steamed pumpkin, compared to raw pumpkin shows that there was over 100% retention of PVACs after cooking. The higher PVACs retention observed in this current study is consistent with other studies conducted in other PVACs‐rich foods such as provitamin A biofortified maize, provitamin A biofortified cassava and orange‐fleshed sweet potato, which showed that PVACs retention ranged from 90% to over 100% with either boiling or steaming (Pillay, Siwela, Derera, & Veldman, 2011; Thakkar, Huo, Maziya‐Dixon, & Failla, 2009; Vimala, Nambisan, & Hariprakash, 2011). Furthermore, these findings are in agreement with previous PVACs retention studies that used boiled or steamed pumpkin (Carvalho et al., 2014, 2015). Carvalho and colleagues demonstrated that the retention of β‐and α‐carotene in both provitamin A biofortified and nonbiofortified pumpkin was over 100% after boiling or steaming (Carvalho et al., 2014, 2015).

The greater than 100% PVACs retention observed in this present study is plausible probably because of the PVACs isomerism characterized by extraction and release of *cis‐*β‐carotene isomers during heating of PVACs‐rich foods (Azizah et al., 2009; Bechoff et al., 2017; Bengtsson et al., 2008; Mugode et al., 2014). Furthermore, maceration (softening) and heating increases the PVACs concentration by rupturing the microstructure of PVACs‐rich plant tissue and then releasing more PVACs from the complex food matrix (Provesi & Amante, 2015; Tumuhimbise, Namutebi, & Muyonga, 2009).

It is worth noting that the higher concentrations of PVACs observed in steamed compared to boiled pumpkin could be attributed to the cooking method. It is likely that in the boiling method, PVACs released by rupturing the microstructure of the plant tissue are drained into the boiling water used to cook the PVACs‐rich food (Provesi & Amante, 2015; Tumuhimbise et al., 2009). Moreover, this present study did not analyze the PVACs content of the water in which the pumpkin was boiled because the water was discarded. The greater than 100% retention of PVACs (β‐carotene and α‐carotene) observed after cooking pumpkin also led to the increase of RAE in boiled and steamed pumpkin. This is because the RAE calculation is based on the quantity of PVACs (Institute of Medicine, 2001). To this end, an increase in PVACs retention increases RAE. Such an increase in RAE has implications in meeting the recommended dietary allowance (RDA) for vitamin A of a target population group such as Ugandan children, 6 to 24 months old, an age group vulnerable to VAD (Amaral et al., 2018; Ekesa et al., 2019). The RDA is the intake of a nutrient that meets the needs of almost all (97% to 98%) individuals in a group (Institute of Medicine, 2001).

### 4.2 Zinc and iron retention in common bean

Iron and zinc content in cooked common bean were lower than in raw common bean, indicating that boiling with or without prior soaking of common bean reduces its iron and zinc retention. These findings are consistent with previous studies by Carvalho et al. (2012), Ferreira et al. (2014) and Hummel et al. (2020), who demonstrated that the zinc and iron retention for boiled common bean with or without soaking is below 100%. However, this current study showed that iron retention was higher in boiled common bean with prior soaking (92.2%) compared to common bean without soaking (88.4%).

Furthermore, zinc retention was higher in boiled common bean with prior soaking (88.4%) compared to common bean without prior soaking (75.6%). Findings from this current study are consistent with other studies, which demonstrated that iron and zinc retention in boiled common bean with prior soaking is higher than boiled common bean without prior soaking (Carvalho et al., 2012; Hummel et al., 2020). For example, in Brazil, Carvalho et al. (2012) showed that iron retention was 99% and 94% for boiled common bean (*BRS marfim*) with prior soaking and boiled common bean (*BRS marfim*) without prior soaking, respectively. Furthermore, Carvalho et al. (2012) demonstrated that zinc retention was higher (84%) in boiled common bean (*BRS Radiante)* with prior soaking compared to boiled common bean *(BRS Radiante)* without prior soaking (78%). Hummel et al. (2020) reported consistent findings from South American and Eastern African cultivated common bean varieties by demonstrating that the mean iron true retention (87%) for boiled common bean with prior soaking was higher compared to boiled common bean without prior soaking among iron biofortified common bean varieties. In contrast to iron, Hummel et al. (2020) showed that mean zinc retention was higher in boiled without prior soaking (81%), compared to boiled with prior soaking (78%) in zinc biofortified common bean varieties. However, there was no statistically significant difference in zinc retention between boiled with or without prior soaking among the zinc biofortified common bean varieties (Hummel et al., 2020).

It is worth noting that previous studies have showed that it is not unanimous that boiled common beans with prior soaking have a higher retention of iron and zinc than those boiled without prior soaking (Carvalho et al., 2012; Fernandes, Nishida, & Da‐Costa Proença, 2010; Hummel et al., 2020). Several other common bean varieties from Eastern African countries and South American countries such as Brazil, and Columbia, boiled with or without prior soaking, had a varied retention of zinc and iron, ranging from 90% to over 100% (Carvalho et al., 2012; Ferreira et al., 2014; Hummel et al., 2020). These findings were not unanimous, since zinc and iron content and retention was higher in some boiled common bean varieties without prior soaking, compared to those boiled with prior soaking (Carvalho et al., 2012; Ferreira et al., 2014; Hummel et al., 2020). This may indicate that the variety of common bean and the environment in which they are cultivated are possible determinants of iron and zinc content and retention. For example, iron and zinc biofortified varieties of common bean cultivated in different countries of South America and East Africa had different iron and zinc content and retention irrespective of either boiling with or without soaking, compared to the noniron/zinc biofortified common bean varieties (Ferreira et al., 2014; Hummel et al., 2020).

The mean iron content of raw nonbiofortified common bean was lower at 7.8 mg/100 g compared to the iron biofortified common bean varieties with mean iron content of 8.9 mg/100 g as reported by Hummel et al. (2020). However, the average iron content of nonbiofortified common bean varieties used by Hummel et al. (2020) was lower at 5.7 g/100 g, compared to that observed in common bean of 7.8 mg/100 g from this present study. Similarly, the zinc biofortified common bean varieties used by Hummel et al. (2020) had higher zinc content at 3.9 mg/100 g, compared to 3 mg/100 g observed in common bean used in the present study. In contrast, the mean zinc content of common bean observed in this current study is almost similar to 3.1 mg/100 g of the nonbiofortified varieties used by Hummel et al. (2020). It is not surprising that biofortified common bean varieties are richer in the micronutrients under study because biofortification is a process that increases the nutritional value of food crops by increasing the density of vitamins and minerals in a crop through either conventional plant breeding, agronomic practices or biotechnology (HarvestPlus, 2007, 2012).

Hummel et al. (2020) boiled common bean with prior soaking, which is similar to the cooking method used in the current study. However, the TR of iron (92%) observed in boiling common bean, *Obwelu* prior to soaking was slightly higher, compared to that of 87% and 88% in iron biofortified and nonbiofortified varieties of common bean, respectively as reported by Hummel et al. (2020). Furthermore, the TR for zinc (91%) observed in soaked boiled *Obwelu* was higher than that of zinc biofortified (80%) and nonzinc biofortified (75%) varieties of common bean used by Hummel et al. (2020), respectively. It is worth noting that a retention of over 90% of iron and zinc observed in boiled common bean with prior soaking in this present study is considered to be high (Carvalho et al., 2012; Ferreira et al., 2014). The higher retention of iron and zinc in boiled common bean with prior soaking compared to boiled common bean without prior soaking, observed in this present study, could be explained by soaking. This is because soaking is more effective in increasing the extractability of iron and zinc from the food matrix, compared to without soaking (Fernandes et al., 2010; Perlas & Gibson, 2002; Sripriya, Antony, & Chandra, 1997).

Furthermore, the water used to soak *Obwelu* was discarded prior to boiling. This is plausible because discarding water after soaking is necessary to reduce phytic acid, an antinutrient that reduces the bioavailability of iron and zinc (Fernandes et al., 2010). In contrast to increasing iron and zinc extractability (Fernandes et al., 2010), soaking may lead to a loss of negligible amounts of iron and zinc loss, because iron and zinc may be lost in the discarded water used for soaking and boiling common bean (Fernandes et al., 2010). This may explain why the retention of iron and zinc was not 100% in soaked, boiled *Obwelu*.

In addition to a higher nutrient retention, soaked common bean cooked faster (1.5 hr) compared to unsoaked common bean (3.5 hr). This finding indicates that boiling with prior soaking of common bean was more fuel saving compared to boiling without prior soaking. This is consistent with a review of previous studies, which confirms that boiling common bean with prior soaking cooks for less time compared to common beans boiled without prior soaking (Reyes‐Moreno, Paredes‐López, & Gonzalenz, 1993). A long cooking time for common bean increases fuel costs as indicated by the higher quantity of charcoal used to cook common bean in the current study (Mendum & Njenga, 2018). It is worth noting that caregivers prefer cooking methods that save time and subsequently save fuel (Reyes‐Moreno et al., 1993). To this end, caregivers would choose a cooking method such as boiling with prior soaking of common beans because it cooks faster, and consequently saves fuel, compared to boiling without prior soaking as demonstrated in this present study.

### 4.3 Strengths and limitations

Some strengths are inherent in the present study. For example, caregivers from the local community prepared pumpkin and common bean by using the locally acceptable home cooking methods. This was important because the locally acceptable cooking methods, which led to higher retention of the micronutrients under study can be promoted and supported through community nutrition education programs (FAO, 2017). However, some limitations also exist. For example, boiling and steaming time impacts differently on PVACs retention in pumpkin (Ribeiro, et al., 2015). However, in this current study, boiling and steaming time for pumpkin were limited to 15 and 30 min, respectively.

Furthermore, iron and zinc retention in common bean are affected differently by different periods of soaking and cooking time (Fernandes et al., 2010). However, the current study used only overnight soaking (8  hr), prior to boiling common bean. It is important to note that it was difficult for this present study to compare micronutrient retention in relation to cooking time for both common bean and pumpkin, since it was not feasible to set a constant cooking temperature when using local cooking equipment such as a charcoal stove used in the current study. It is worth noting that local cooking equipment such as charcoal and firewood stoves commonly used to cook food in developing countries lack temperature regulators, compared to electric cookers commonly used in developed countries (Maes & Verbist, 2012). Furthermore, the three forms of PVACs are β‐carotene, α‐carotene, and β‐cryptoxanthin (Institute of Medicine, 2001). However, the current study did not analyze for β‐cryptoxanthin. Nevertheless, failure to analyze β‐cryptoxanthin may not have significantly affected the PVACs content of pumpkin because evidence from previous studies that analyzed β‐cryptoxanthin in pumpkin, established that its content was either not detected or negligible (Kim et al., 2012).

## 5 CONCLUSIONS

To retain a high proportion of PVACs caregivers should be advised to cook pumpkin, *Sweet cream* by either boiling or steaming, while to retain a high proportion of iron and zinc, common bean, *Obwelu* should be prepared by boiling with prior soaking.

## AUTHOR CONTRIBUTIONS

Conceptualization, E.B.; Methodology, E.B., K.P. and M.S.; Resources, E.B; K.P. and M.S.; Data analysis and interpretation, E.B.; Writing‐original draft preparation, E.B.; Writing‐review and editing, E.B., K.P. and M.S.; Supervision, K.P. and M.S. All authors have read and agreed to the published version of the manuscript.

## ETHICAL STATEMENT

This study does not involve any human or animal testing. The Biomedical Research Ethical Committee, University of KwaZulu‐Natal, South Africa (Reference number: BE 438/19) and the AIDS Support Organisation Research Ethical Committee, Uganda (Reference number: TASO‐REC/066/19‐UG‐REC‐009) approved this study. The authors declare that they have no conflict of interest.
